# Dataset of foraminiferal sedimentary DNA (*sed*DNA) sequences from Svalbard

**DOI:** 10.1016/j.dib.2020.105553

**Published:** 2020-04-18

**Authors:** Joanna Pawłowska, Jan Pawlowski, Marek Zajączkowski

**Affiliations:** a Institute of Oceanology Polish Academy of Sciences, Sopot 81-712, Poland; b Department of Genetics and Evolution, University of Geneva, Geneva CH 1211, Switzerland

**Keywords:** Metabarcoding, sedimentary DNA, Environmental DNA, Foraminifera, Svalbard

## Abstract

Environmental DNA (*e*DNA) is usually defined as genetic material obtained directly from environmental samples, such as soil, water, or ice. Coupled to DNA metabarcoding, *e*DNA is a powerful tool in biodiversity assessments. Results from eDNA approach provided valuable insights to the studies of past and contemporary biodiversity in terrestrial and aquatic environments. However, the state and fate of *e*DNA are still investigated and the knowledge about the form of *e*DNA (i.e., extracellular vs. intracellular) or the DNA degradation under different environmental conditions is limited. Here, we tackle this issue by analyzing foraminiferal sedimentary DNA (*sed*DNA) from different size fractions of marine sediments: >500 µm, 500–100 µm, 100–63 µm, and < 63 µm. Surface sediment samples were collected at 15 sampling stations located in the Svalbard archipelago. Sequences of the foraminifera-specific 37f region were generated using Illumina technology. The presented data may be used as a reference for a wide range of *e*DNA-based studies, including biomonitoring and biodiversity assessments across time and space.

Specification table

**Table:** 

Subject	Environmental Science, Oceanography, Molecular Biology,
Specific subject area	Foraminifera biodiversity and spatial distribution
Type of data	Tables
How data were acquired	Amplicon high-throughput sequencing with Illumina technology. SLIM pipeline to produce OTU-to-sample tables.
Data format	Analyzed data
Parameters for data collection	Surface sediment samples collected with box corer during the cruise of R/V Oceania in 2016.
Description of data collection	The surface sediment samples were collected at 15 sampling stations located in the Svalbard Archipelago. The total DNA was extracted from sediments, foraminifer-specific DNA fragments were amplified and sequenced according to standard protocols.
Data source location	Institute of Oceanology Polish Academy of Sciences, Sopot, Poland
Data accessibility	Repository name: Mendeley Data
	Data identification number: doi: 10.17632/7kjkf8by5d.1
	Direct URL to data: https://data.mendeley.com/datasets/7kjkf8by5d/1

## Value of the data

• The data provides the first insight into the genetic diversity of Arctic foraminifera in different sediment size fractions.
• Also, it's an overview of the spatial distribution of Arctic foraminifera inferred from *sed*DNA.
• This data may serve as a reference in a wide range of metagenomics-based studies, including biomonitoring, biodiversity surveys, and environmental impact assessment studies.
• The data may be used also in the studies of past climatic and environmental changes.

## 1 Data description

**Fig. 1:**
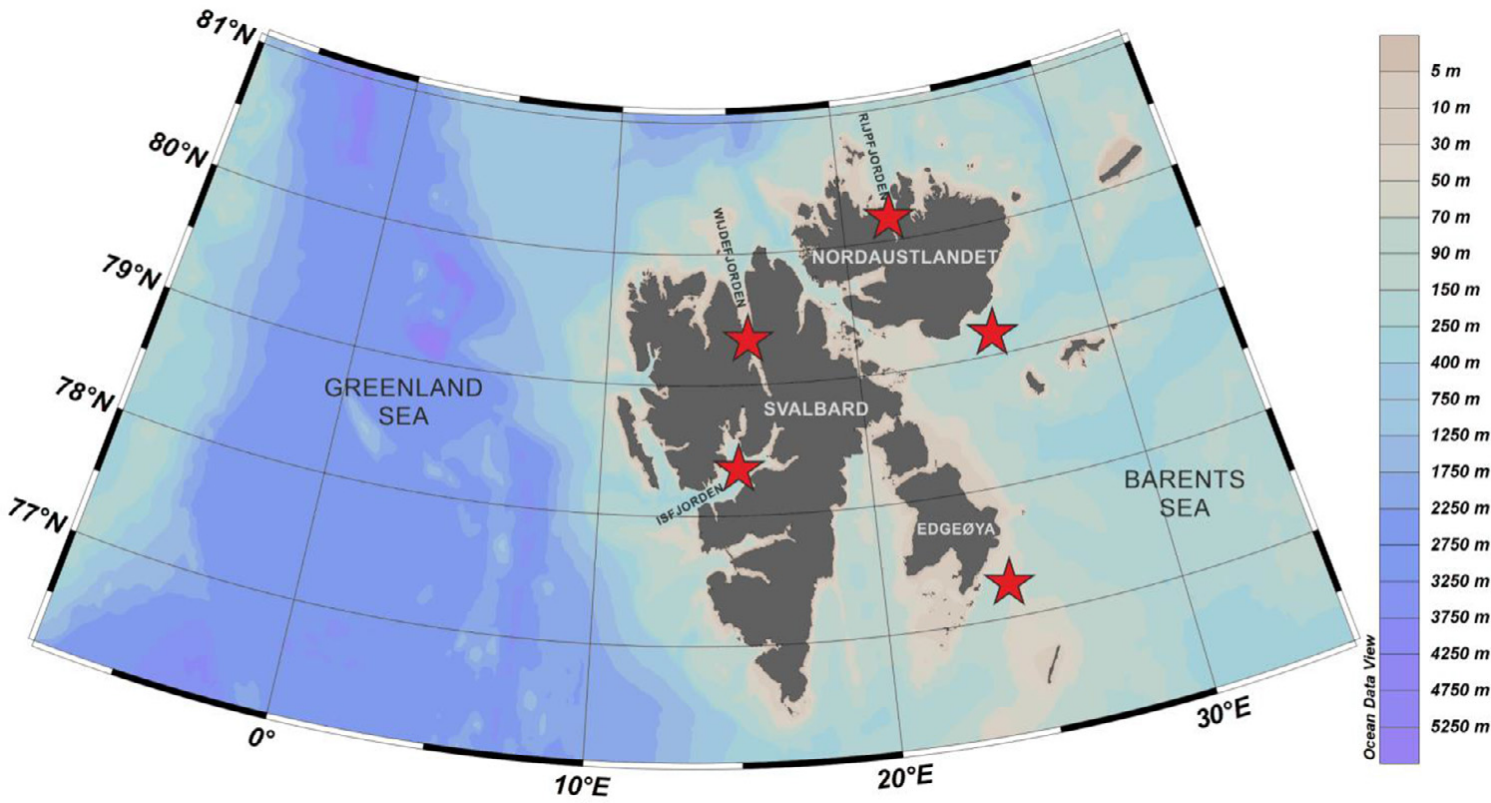
Study area and sampling localities.

**Fig. 2:**
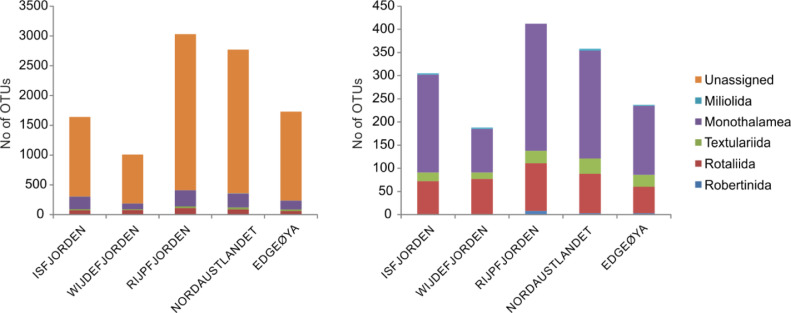
Number of OTUs found in each sampling locality, including unassigned OTUs (left panel) and excluding unassigned OTUs (right panel).

**Fig. 3:**
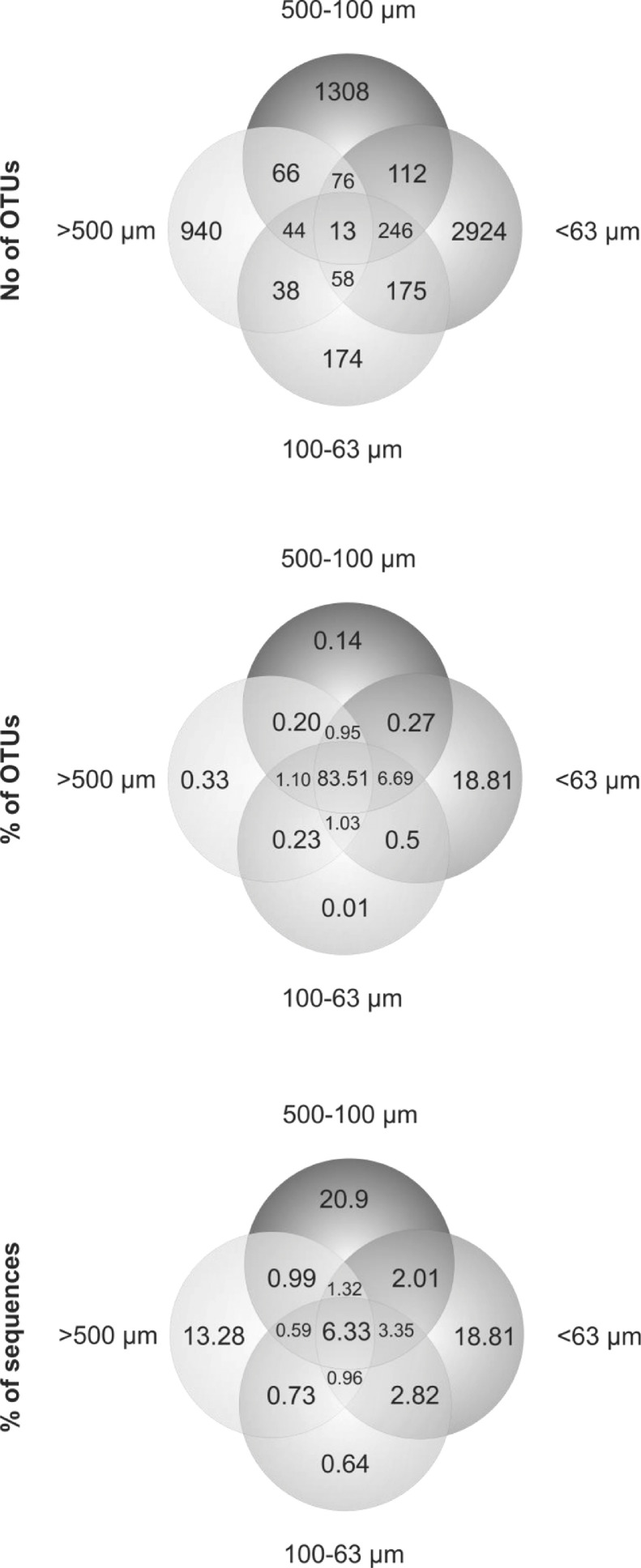
Venn diagrams of number of OTUs, percent [%] of OTUs, and percent [%] of sequences found in the studied sediment fractions (>500 µm, 500–100 µm, 100–63 µm, <63 µm).

The dataset contains foraminiferal *sed*DNA sequences from four sediments size fractions: >500 µm, 500–100 µm, 100–63 µm, and <63 µm. Sequences are clustered into the Operational Taxonomic Units (OTUs), and for each OTU, the number of sequence reads is presented. The data set can be accessed at Mendeley Data (doi: 10.17632/7kjkf8by5d.1). The samples were collected at 16 sampling stations collected from five localities in the Svalbard archipelago (Fig. 1). Sampling stations coordinates and sampling depths can be found in Table 1. The total number of OTUs recorded in each sampling location is presented in Fig. 2. The number and percentage of OTUs and the percentage of DNA sequences found in the certain sediment size fractions are presented on the Venn diagrams (Fig. 3).

**Table 1:** Geographical coordinates and depths of sampling stations.

Station	Depth [m]	Latitude [°N]	Longtitude [°W]
Isfjorden 1	191	78 24.000	15 35.690
Isfjorden 2	240	78 15.890	14 51.200
Wijdefjorden 1	160	79 53.815	15 19.066
Wijdefjorden 3	154	79 09.464	16 00.085
Rijpfjorden 1	202	80 05.374	22 12.966
Rijpfjorden 2	260	80 18.464	22 10.951
Rijpfjorden 3	260	80 22.354	22 05.736
Rijpfjorden 4	155	80 30.288	22 02.996
Nordaustlandet 4	42	79 42.727	26 34.955
Nordaustlandet 5	82	79 40.506	26 48.952
Nordaustlandet 6	147	79 36.241	27 30.196
Nordaustlandet 7	275	79 33.185	28 00.347
Edgeøya 1	41	77 42.925	24 12.853
Edgeøya 2	71	77 41.147	24 39.384
Edgeøya 3	114	77 39.751	25 00.162

## 2 Experimental design, materials, and methods

### 2.1 Sampling

Surface sediment samples were collected with the use of box corer during the cruise of R/V Oceania in August 2016. The upper 2 cm of sediment has been sampled from the surface of approximately 25 cm^2^. Samples for sedimentary DNA (*sed*DNA) analysis were wet sieved on a 500 µm, 100 µm, and 63 µm sieves. A fraction smaller than 63 µm was retained. Samples were transferred to sterile containers and froze in −20 °C.

### 2.2 *sed*DNA analysis

The DNA from sediment fractions 500 µm, 100 µm, and 63 µm was extracted from 0.25 g of bulk sediment with DNeasy PowerSoil Kit (Qiagen). Due to a large amount of sediment in <63 µm fraction, DNA was extracted from 10 g of sediment using DNeasy PowerMax Soil Kit (Qiagen). The SSU DNA fragment including foraminifera-specific 37f hypervariable region [1] has been PCR amplified with the s14F1 (5′-XXXXXAAGGGCACCACAAGAACGC-3′) and s15 (5′-XXXXXCCTATCACATAATCATGAAAG-3′) primers tagged with unique sequences of 5 nucleotides appended at their 5′ ends. For each sample, 3 PCR replicates were prepared. Amplicons were quantified with Qubit 3.0 fluorometer and the pool was purified with High PCR Cleanup Micro Kit (Roche). Library preparation was performed with TruSeq DNA PCR-Free LT Library Prep Kit (Illumina) and was loaded onto a MiSeq instrument for a paired-end HTS run of 2 × 150 cycles.

### 2.3 Post-sequencing data processing

Raw sequence data were processed according to [2,3]. The post-sequencing data processing was performed using the SLIM web app [4] and included demultiplexing the libraries, joining the paired-end reads, chimera removal, Operational Taxonomic Units (OTUs) clustering, and taxonomic assignment. Sequences were clustered into OTUs using the Swarm module [5] and each OTU was assigned to the highest possible taxonomic level using vsearch [6] against a local database of foraminiferal SSU DNA sequences. The results were presented as OTUs-to-samples tables.

## Appendix Supplementary materials

Image, application 1
